# A Novel Molecularly Imprinted Quartz Crystal Microbalance Sensor Based on Erbium Molybdate Incorporating Sulfur-Doped Graphitic Carbon Nitride for Dimethoate Determination in Apple Juice Samples

**DOI:** 10.3390/foods13050810

**Published:** 2024-03-06

**Authors:** Neslihan Özdemir, Betül Karslıoğlu, Bahar Bankoğlu Yola, Necip Atar, Mehmet Lütfi Yola

**Affiliations:** 1 Department of Machinery and Metal Technologies, Merzifon Vocational School, Amasya University, Amasya 05300, Turkey; neslihan.ozdemir@amasya.edu.tr; 2 Department of Gastronomy and Culinary Arts, Faculty of Tourism, Hasan Kalyoncu University, Gaziantep 27000, Turkey; betul.kokay@hku.edu.tr; 3 Department of Engineering Basic Sciences, Faculty of Engineering and Natural Sciences, Gaziantep Islam Science and Technology University, Gaziantep 27000, Turkey; bahar.bankogluyola@gibtu.edu.tr; 4 Department of Chemical Engineering, Faculty of Engineering, Pamukkale University, Denizli 20160, Turkey; natar@pau.edu.tr; 5 Department of Nutrition and Dietetics, Faculty of Health Sciences, Hasan Kalyoncu University, Gaziantep 27000, Turkey

**Keywords:** dimethoate, quartz crystal microbalance, nanocomposite, apple juice sample

## Abstract

Dimethoate (DIM) as an organophosphorus pesticide is widely utilized especially in the cultivation of vegetables and fruits due to its killing effect on harmful insects. However, unconscious use of DIM in large amounts can also cause serious health problems. For these reasons, rapid and reliable detection of DIM from food samples is significant. In this study, a novel quartz crystal microbalance (QCM) sensor based on erbium molybdate incorporating sulfur-doped graphitic carbon nitride (EM/S-g-C_3_N_4_) and a molecularly imprinting polymer (MIP) was designed for DIM detection in apple juice samples. Firstly, an EM/S-g-C_3_N_4_ nanocomposite with high purity was prepared under hydrothermal conditions at high temperatures over a long period of time. After the modification of the EM/S-g-C_3_N_4_ nanocomposite on a QCM chip, the polymerization solution including N,N′-azobisisobutyronitrile (AIBN) as an initiator, ethylene glycol dimethacrylate (EGDMA) as a cross-linker, methacryloylamidoglutamic acid (MAGA) as a monomer, and DIM as an analyte was prepared. Then, the polymerization solution was dropped on an EM/S-g-C_3_N_4_ nanocomposite modified QCM chip and an ultraviolet polymerization process was applied for the formation of the DIM-imprinted polymers on the EM/S-g-C_3_N_4_ nanocomposite modified QCM chip. After the polymerization treatment, some characterization studies, including electrochemical, microscopic, and spectroscopic methods, were performed to illuminate the surface properties of the nanocomposite and the prepared QCM sensor. The values of the limit of quantification (LOQ) and the detection limit (LOD) of the prepared QCM sensor were as 1.0 × 10^−9^ M and 3.3 × 10^−10^ M, respectively. In addition, high selectivity, stability, reproducibility, and repeatability of the developed sensor was observed, providing highly reliable analysis results. Finally, thanks to the prepared sensor, it may be possible to detect pesticides from different food and environmental samples in the future.

## 1. Introduction

Apples are one of the most traded fruits in the world [1]. Apples, as a food source, provide a high amount of bioactive compounds and are significant sources of flavonoids, and are particularly rich in the flavonol quercetin and its derivatives [2,3]. In addition to being enjoyed fresh, apples can be processed into various apple products, such as apple juices, dehydrated apples, and apple purées, using different processing technologies [4,5]. The most consumed among these products is apple juice, and it is also holds significance in complementary foods for infants and children [6]. Despite the undeniable role of fruit juices on health, pollutants, especially from agricultural sources such as pesticides, can be a risk for juices [7,8]. Due to their persistence, long-distance transport, biological effects, and accumulation along the food chain, pesticide contamination is among the most concerning issues. Therefore, monitoring and detecting the presence of these compounds in the environment, especially in food samples, is of great importance for both human health and environmental control [9]. Numerous studies have indicated that the presence of pesticide residues in apples could pose safety risks to human health, potentially causing DNA damage and inducing oxidative stress through various mechanisms [10,11,12].

DIM, an organophosphorus pesticide widely used in apple crops, is highly effective as a broad-spectrum acaricide [13]. Its acceptable value for daily intake is 0.002 mg/kg body weight day [14]. The most concerning aspect of human exposure to this acaricide is that it leads to acetylcholine accumulation in the body [15]. The presence of DIM residues in food may lead to severe health issues, such as depression, anxiety, and irritability. Furthermore, prolonged exposure to high levels may contribute to the development of conditions such as anemia and cancer [16,17,18]. Considering the adverse effects of DIM residues on health, it is crucial to develop rapid, convenient, and sensitive methods for their detection in apple juices. Some detection methods, including the colorimetric method, immunoassays, chromatography, chromatography-mass spectrometry, and high-performance liquid chromatography-coupled tandem mass spectrometry (LC-MS/MS) have been used for the detection of DIM residues [14,15,19,20]. While these methods can accurately determine low concentrations of DIM, their accuracy, selectivity, and precision are generally negatively affected by complex food matrices [21]. Therefore, due to QCM sensors’ high selectivity, repeatability, and selectivity properties, the sensor systems having the piezoelectric effect, such as QCM, are significant to ensure the reliability of food consumption.

Metal molybdates with different shapes and geometries including nanoplates, flower-like mesocrystals, and nanorods are found in the literature [22,23,24]. Metal molybdates have started to attract attention in some applications including electrocatalysis and photocatalysis. For example, a nanocomposite including cerium molybdate and graphene oxide was prepared for the photo-degradation of the drug chloramphenicol [25]. In another study, a nanocomposite including graphitic carbon nitride nanosheets and lanthanum molybdate was prepared for the photo-degradation of tetracycline [26]. Hence, the rare-earth elements have been effective catalysts due to optical and electrochemical properties [26]. In addition, lanthanide metal ions can efficiently undergo complexation reactions with different types of Lewis bases, resulting in further improvements in their electrical and optical properties [27]. Moreover, erbium (Er) as a dopant is one of the important lanthanides and can provide particle diameter reduction, causing increased catalytic activity and surface area improvement [28].

Carbon-based materials as heterogeneous catalysts have been widely utilized owing to their large surface areas, biocompatibility, and metal-free features. Especially notable is S-g-C_3_N_4_, with band-gap of 2.7 eV and its layered polymeric networks, carbon with sp^2^ hybridization, and nitrogen atoms [29]. Its preparation can be performed by using a facile synthetic method and has superior stability compared to acidic/alkaline media [30]. In addition, the catalytic efficiency of S-g-C_3_N_4_ can be increased by various methods such as the preparation of S-g-C_3_N_4_-based heterojunction structures, doping with metals or nonmetals, and structural modifications [31,32]. Molecular imprinting is an accepted approach to produce template molecule-directed, highly selective polymeric materials. This method proposes the recognition of molecules with certain nano-cavities with compatibility in terms of structure, size, and chemical functions. Molecular recognition is based on the complexation between receptor and substrate. This definition is stated as the “Key–Lock” model in the literature. According to this theory, intermolecular interactions are regulated in parallel with the compatibility between complementary functional groups, such as between enzyme and substrate or antibody and antigen. In the molecular imprinting method, suitable monomers, cross-linkers, and the target molecule are present together. In the solution environment, monomers and cross-linkers are arranged around the target molecule with the support of forces such as electrostatic forces, dipolar interactions, hydrogen bonding, Wan der Waals interactions, or π-π interactions. In the next stage, macromolecular materials emerge with the interaction network stabilized as a result of the polymerization reaction. After the polymerization process, the target molecule is removed together with the appropriate desorption agent. As a result, nano-cavities are formed in the structure that contain the steric and functional properties of the molecule [33,34].

In this study, we present the design of a sensitive molecularly imprinted QCM sensor based on erbium molybdate incorporating sulfur-doped graphitic carbon nitride. The nanocomposite demonstrated a better QCM sensor catalytic efficiency in comparison with erbium molybdate and sulfur-doped graphitic carbon nitride. The newly designed QCM sensor demonstrated effective application in apple juice samples, displaying high recovery rates and selectivity. Thus, with this developed QCM sensor, it becomes possible to detect important health risks associated with pesticide exposure in advance.

## 2. Materials and Methods

### 2.1. Materials

DIM, acephate (ACE), trichlorfon (TRI), hexachlorocyclohexane (HEX), fenvalerate (FEN), dichlorodiphenyltrichloroethane (DDT), thiourea (THI), erbium nitrate (ErH_10_N_3_O_15_), sodium molybdate (Na_2_MoO_4_·2H_2_O), AIBN, EGDMA, MAGA, hydroxyethylmethacrylate (HEMA), and sodium chloride (NaCl) were obtained from Sigma-Aldrich Merck Group company (St. Louis, MO, USA). Phosphate-buffered saline (0.1 M PBS with a pH of 6.0) was used for dilution solution.

### 2.2. Instrumentation

The analytical techniques employed for structural characterization studies are outlined in the Supplementary Materials. Additionally, INFICON Acquires Maxtek (New York, NY, USA) was utilized for QCM analytical measurements.

### 2.3. Synthesis of EM, S-g-C_3_N_4_, and the EM/S-g-C_3_N_4_ Nanocomposite 

The preparation of EM was completed by the hydrothermal method including ErH_10_N_3_O_15_ solution (0.04 M, 25.0 mL) and Na_2_MoO_4_·2H_2_O solution (0.02 M, 25.0 mL). After this dispersion was firstly sonicated for 15 min, NaOH solution (1.0 M) was slowly dropped into this dispersion until pH 9.0. Then, the heating treatment was applied to the dispersion at 150 °C for 10 h into a stainless autoclave. After the cooling treatment to 25 °C, the pink EM product was washed three times with ethanol and then with ultra-deionized water and stored at room temperature. Finally, the obtained pink EM product was calcined for 5 h at 750 °C, providing flower-like structures [35]. The preparation of S-g-C_3_N_4_ was carried out by the pyrolysis treatment in the presence of THI (15.0 g) at 600 °C for 4 h, according to our previous paper [36].

The preparation of the EM/S-g-C_3_N_4_ nanocomposite was performed by using a facile grinding technique. The mixture of EM and S-g-C_3_N_4_ (1:1 wt%) was ground for 20 min, suggesting the EM/S-g-C_3_N_4_ nanocomposite.

### 2.4. QCM Chip Modification with EM/S-g-C_3_N_4_ and the Production of a DIM-Imprinted QCM Sensor Based on EM/S-g-C_3_N_4_

Before analytical applications and modification procedures, QCM chips were cleaned by interacting with acidic piranha solution (15.0 mL, (3:1) H_2_SO_4_:H_2_O_2_, *v*/*v*) in a shaking bath system for 15 min. After 15 min, QCM chips were washed three times with ultra-pure distilled water for 30 min and then dried at room temperature to become ready for use. The modification treatment was performed by dropping EM/S-g-C_3_N_4_ solution (25.0 µL, 25.0 mg/mL) on QCM chips via the high affinity between gold and sulfur (EM/S-g-C_3_N_4_/QCM) [37].

For the preparation of DIM-imprinted QCM sensors based on EM/S-g-C_3_N_4_, firstly, a MAGA-DIM complex (5.0 mL) was prepared in the stoichiometric ratio of (1:2) in the presence of pH 6.0 PBS. Then, the polymerization solution including EGDMA (4.0 mL), AIBN (20.0 mg), and HEMA (2.0 mL) was prepared in a shaking bath system (10.0 mL). After slowly adding the complex solution to the polymerization solution during 30 min under mixing treatment, the final solution (5.0 mL) was dropped on the EM/S-g-C_3_N_4_/QCM via the spin coating method. After 1 min, the drying process of the QCM chip was conducted at 25 °C and the ultraviolet polymerization process was applied, suggesting a DIM-imprinted QCM sensor based on EM/S-g-C_3_N_4_ (MIP/EM/S-g-C_3_N_4_/QCM). To illustrate the imprinting selectivity, a DIM non-imprinted QCM sensor based on EM/S-g-C_3_N_4_ (NIP/EM/S-g-C_3_N_4_/QCM) was prepared via the same procedure above without DIM analyte.

### 2.5. DIM Removal and Analysis Procedure

In this study, 0.1 M NaCl was used as a desorption solution to break the electrostatic interactions between the monomer and the analyte molecule in order to remove the DIM analyte imprinted on the QCM chip surface from the chip surface. For this aim, the MIP/EM/S-g-C_3_N_4_/QCM chip was placed in a conical flask containing NaCl solution (0.1 M, 10.0 mL) and activated for 10 min (desorption time). After 10 min, DIM-removed QCM chips were dried at 25 °C and placed in the QCM cell.

In the DIM analysis procedure, firstly, 0.1 M PBS (pH 6.0) solution with 2.0 mL/min flow-rate was transferred into a QCM cell to provide the QCM system equilibration in 10 min. Secondly, adsorption experiments were performed using increasing concentrations of DIM standard solutions over 10 to 50 min. After completing the desorption part of DIM solutions at each concentration with 0.1 M NaCl solution with 2.0 mL/min flow-rate, the equilibration was conducted with 0.1 M PBS (pH 6.0) in the last 10 min (regeneration part).

### 2.6. Sample Preparation

Firstly, 10.0 mL of the apple juice sample obtained from the local market was taken and transferred to a volumetric flask (25.0 mL). After the sample was centrifuged for 5 min to ensure homogenization, the dilution was performed with 0.1 M PBS (pH 6.0) to fall within the DIM linearity range and the apple juice sample was transferred with 2.0 mL min^−1^ flow-rate into the QCM cell.

## 3. Results and Discussion

### 3.1. Characterizations of EM, S-g-C_3_N_4_, and the EM/S-g-C_3_N_4_ Nanocomposite

**Figure 1:**
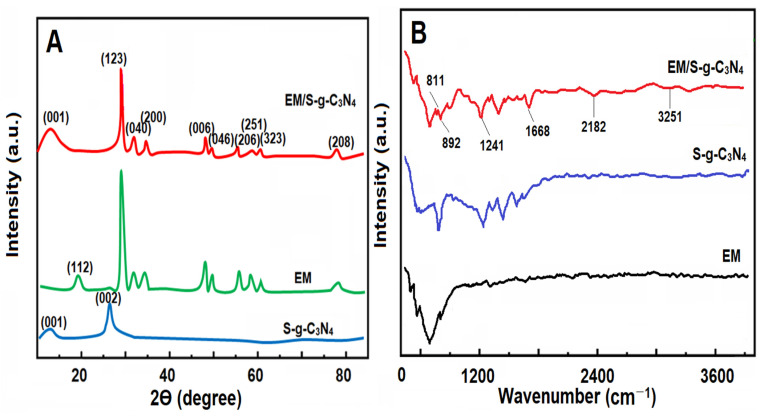
(**A**) XRD pattern and (**B**) FTIR spectra of the EM/S-g-C_3_N_4_ nanocomposite, EM, and S-g-C_3_N_4_.

To highlight the structural features of the synthesized materials including the EM/S-g-C_3_N_4_ nanocomposite, EM, and S-g-C_3_N_4_, XRD measurements were firstly performed (Figure 1A). Two XRD peaks at 13.31° and 28.11° corresponded to (001) and (002) planes, respectively, for S-g-C_3_N_4_ [38]. EM demonstrated several XRD peaks at 19.69°, 30.08°, 32.94°, 33.38°, 35.08°, 48.07°, 48.41°, 50.14°, 56.09°, 58.94°, 60.71°, 74.21°, and 79.07°, attributing to (112), (123), (040), (200), (006), (046), (206), (251), (323), and (208) planes, respectively. Finally, the presence of XRD peaks relating to EM and S-g-C_3_N_4_ in the nanocomposite verified the successful synthesis of the EM/S-g-C_3_N_4_ nanocomposite without any impurities [39]. FTIR spectra (Figure 1B) were obtained for the EM/S-g-C_3_N_4_ nanocomposite, EM, and S-g-C_3_N_4_. In the absorption spectrum of EM, the absorption bands between 3000 and 3600 cm^−1^ were attributed to O–H stretching and H–O–H bending, providing the presence of the coordinated H_2_O molecules. In addition, two stretching and two bending bands corresponding to the free tetrahedral MoO_4_^2−^ were observed at 690, 1420, and 515 cm^−1^ [40]. In the absorption spectrum of S-g-C_3_N_4_, the absorption bands at 811 and 892 cm^−1^ were related to the triazine units’ breathing modes in CN heterocycles and N–H deformation, respectively [41]. In addition, the absorption bands between 1241 and 1668 cm^−1^ corresponded to heptazine heterocyclic rings belonging to g-C_3_N_4_ [35]. The weak absorption bands at about 2182 and 3251 cm^−1^ were attributed to C–C groups’ valence vibrations and H_2_O molecule/amine structure including –OH/–NH groups, respectively. It could be seen that the absorption peaks appearing in the absorption spectra of EM and S-g-C_3_N_4_ appeared in the spectrum of the nanocomposite. 

**Figure 2:**
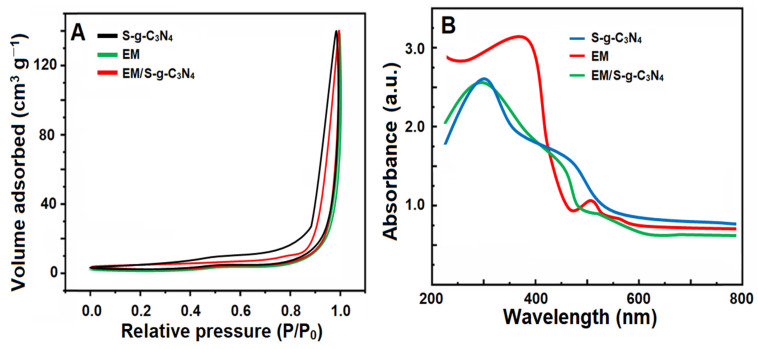
(**A**) N_2_ adsorption/desorption isotherms and (**B**) UV-Vis spectra of the EM/S-g-C_3_N_4_ nanocomposite, EM, and S-g-C_3_N_4_.

N_2_ adsorption/desorption isotherms were provided for the determination of the surface area and porous characteristics of the EM/S-g-C_3_N_4_ nanocomposite, EM, and S-g-C_3_N_4_ (Figure 2A). According to Figure 2A, the EM/S-g-C_3_N_4_ nanocomposite, EM, and S-g-C_3_N_4_ exhibited H3 hysteresis loops, providing the internal porosity with slit and panel shapes. BET surface area values of EM, S-g-C_3_N_4_, and the EM/S-g-C_3_N_4_ nanocomposite were obtained to be 9.27, 14.37, and 19.71 m^2^/g, respectively. Thus, the incorporation of S-g-C_3_N_4_ into EM significantly increased the surface area. Moreover, optical measurements were provided to obtain the physical features of the EM/S-g-C_3_N_4_ nanocomposite, EM, and S-g-C_3_N_4_ (Figure 2B). The absorbance of S-g-C_3_N_4_ at about 428 nm suggested a band gap transition resulting from visible light absorption. In addition, the higher absorbance of EM at about 392 nm was observed. Lastly, the absorbance of the EM/S-g-C_3_N_4_ nanocomposite was relatively higher than EM and S-g-C_3_N_4_, showing a strong absorption ability causing catalytic performance. 

**Figure 3:**
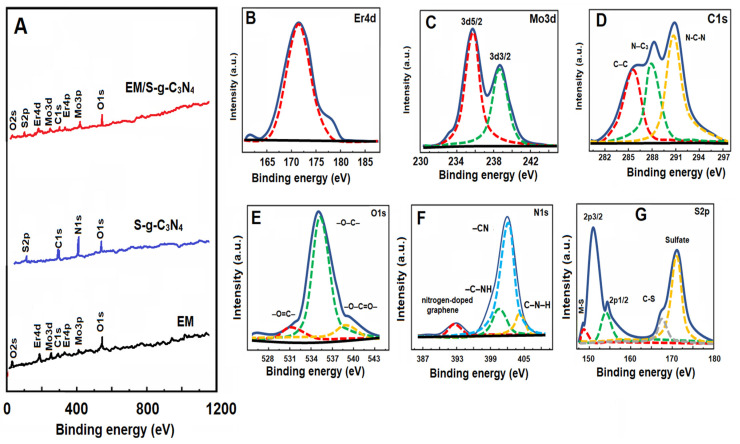
Survey spectra of (**A**) the EM/S-g-C_3_N_4_ nanocomposite, EM, and S-g-C_3_N_4_ and high-resolution XPS spectra of the EM/S-g-C_3_N_4_ nanocomposite: (**B**) Er4d, (**C**) Mo3d, (**D**) C1s, (**E**) O1s, (**F**) N1s, and (**G**) S2p.

Survey spectra of the EM/S-g-C_3_N_4_ nanocomposite, EM, and S-g-C_3_N_4_ and high-resolution XPS spectra of Er4d, Mo3d, C1s, O1s, N1s, and S2p are given in Figure 3. According to the survey spectra (Figure 3A), the presence of elemental components making up all three materials verified the formation of the EM/S-g-C_3_N_4_ nanocomposite, EM, and S-g-C_3_N_4_. The XPS peak of Er4d at 171.27 eV confirmed the erbium coupling with metal oxide (Figure 3B) [39]. According to the Mo3d XPS spectrum (Figure 3C), the peaks at 236.02 and 239.16 eV were attributed to Mo^6+^ oxidation corresponding to Mo3d_5/2_ and Mo3d_3/2_, respectively [42]. The C1s XPS peaks at 290.89, 286.34, and 289.37 eV were related to N-C-N including sp^2^-bonded carbon, C–C, and N–C_3_ bonds, respectively (Figure 3D) [43]. Figure 3E shows the O1s XPS spectrum including three peaks at 532.13 eV having an –O=C– group, 536.17 eV having an –O–C– group, and 540.37 eV having an –O–C=O– group, respectively [44]. Four XPS peaks at 393.12, 401.37, 402.84, and 404.31 eV were attributed to nitrogen-doped graphene, –C–NH, –CN, and C–N–H on the N1s spectrum (Figure 3F) [45]. The prominent peaks at 151.37 and 155.46 eV were attributed to the –C–S–C– group on the S2p spectrum. Furthermore, the XPS peak at 149.34 eV was related to the metal–S bond, and the XPS peak at 168.84 indicated a –C–S– bond, suggesting sulfur presence in g-C_3_N_4_ sheets (Figure 3G) [46].

**Figure 4:**
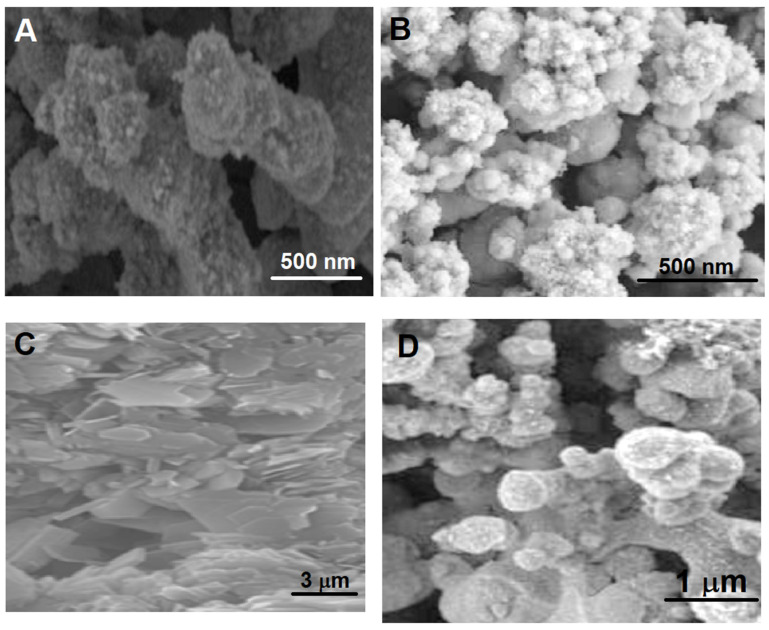
FESEM images of (**A**) EM before calcination, (**B**) EM after calcination, (**C**) S-g-C_3_N_4_, and (**D**) the EM/S-g-C_3_N_4_ nanocomposite.

FESEM images (Figure 4) were recorded to study the surface morphologies of the EM/S-g-C_3_N_4_ nanocomposite, EM, and S-g-C_3_N_4_. According to Figure 4A, the formation of the flower-like nanostructure was incomplete before the calcination treatment. After the calcination treatment, the flower-like structures were obtained (Figure 4B). A two-dimensional nanostructure including smooth surfaces (Figure 4C) was observed on the FESEM image of S-g-C_3_N_4_, and EM particles coupled with S-g-C_3_N_4_ included a two-dimensional nanostructure (Figure 4D).

The interior structural morphology of the EM/S-g-C_3_N_4_ nanocomposite was investigated by TEM and HRTEM images. Figure S1A demonstrated a flower-like EM/S-g-C_3_N_4_ nanocomposite including hetero-interactions between EM and S-g-C_3_N_4_. According to Figure S1B, the lattice fringes including d-spacing values of 0.319 nm were seen, providing the (123) plane. Finally, EDX analysis (Figure S2) of the EM/S-g-C_3_N_4_ nanocomposite showed the existence of erbium, molybdenum, sulfur, carbon, nitrogen, and oxygen. Hence, the successful synthesis of the EM/S-g-C_3_N_4_ nanocomposite was completed.

### 3.2. Electrochemical Characterizations of the EM/S-g-C_3_N_4_ Nanocomposite, EM, and S-g-C_3_N_4_ Modified Electrodes

Electrochemical characterization processes were performed using EIS and CV techniques to understand in detail the electrochemical activity and conductivity comparisons of the prepared electrode materials such as the EM/S-g-C_3_N_4_ nanocomposite, EM, and S-g-C_3_N_4_ (Figure S3A). Initially, the anodic and cathodic peaks were observed at a certain current value using bare GCE (curve a). Owing to erbium molybdate’s increased conductive properties, the more anodic and cathodic peaks were seen on EM/GCE (curve b) [39]. The increased electrochemical signals were observed on S-g-C_3_N_4_/GCE in comparison with EM/GCE because g-C_3_N_4_ had a high number of active sites and monolayers providing a catalytic effect (curve c) [36]. In this study, the highest electro-catalytic effect was obtained using EM/S-g-C_3_N_4_/GCE (curve d) due to the synergistic effect between EM and S-g-C_3_N_4_ and a large electrochemical surface area [35]. The electroactive surface areas of bare GCE, EM/GCE, S-g-C_3_N_4_/GCE, and EM/S-g-C_3_N_4_/GCE were calculated as 0.073 ± 0.005, 0.379 ± 0.003, 0.519 ± 0.004, and 0.937 ± 0.004 cm^2^, respectively, by using the i_p_ = 2.69 × 10^5^ A n^3/2^ D^1/2^ C v^1/2^ equation in the presence of 1.0 mM [Fe(CN)_6_]^3−^. In light of the results obtained from CV and electrode surface calculations, because molybdate-based materials had high surface stabilities and conductivities, as well as the sensor effect of g-C_3_N_4_ nanomaterial, which has graphene-like physical and chemical properties, the EM/S-g-C_3_N_4_ nanocomposite material was used as a sensor surface for subsequent analytical applications.

Moreover, EIS measurements (Figure S3B) were also compared for the electrode conductivities. The charge transfer resistance (R_ct_) values were calculated as 60 ohms for bare GCE (curve a), 45 ohms for EM/GCE (curve b), 38 ohms for S-g-C_3_N_4_/GCE (curve c), and 30 ohms for EM/S-g-C_3_N_4_/GCE (curve d) in harmony with the CV results.

### 3.3. AFM and FTIR Studies of Molecularly Imprinted Film on EM/S-g-C_3_N_4_/QCM

The AFM surface roughness technique was frequently used in recent years for surface analysis of sensor surfaces, especially the QCM sensor. For this aim, AFM images of bare QCM chips (Figure S4A) and molecularly imprinted film on EM/S-g-C_3_N_4_/QCM (Figure S4B) were obtained and the roughness values of 4.33 ± 0.07 and 47.19 ± 0.01 nm were calculated for the bare QCM chip and the modified QCM chip, respectively. In light of these results, it was possible to say that DIM-imprinted QCM chips were successfully produced. Additionally, FTIR measurements (Figure S4C) were performed to prove the presence of the prepared polymerization solution including MAGA and HEMA on the EM/S-g-C_3_N_4_/QCM chip surface. The absorption peaks at 3591, 2991, 1701, and 1438 cm^−1^ indicated the existence of HEMA-MAGA, –CH stretching belonging to MAGA, a carboxyl–carbonyl group, and –COO– stretching, respectively.

### 3.4. pH Effect on MIP/EM/S-g-C_3_N_4_/QCM Signals

**Figure 5:**
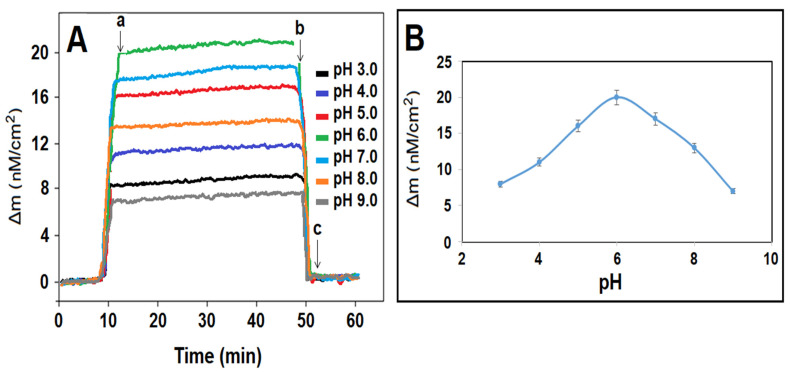
(**A**) QCM sensorgrams with different pHs of PBS for 10.0 nM DIM. (**B**) Effect of pH on QCM signals: (a) adsorption; (b) desorption; (c) regeneration.

Since MAGA, used as a monomer in this study, is a carboxylic acid-based monomer, it had two pKa values (pKa1: 2.10 and pKa2: 4.07). Because the MAGA monomer was easily ionized at different pH values, it easily interacted with the target molecule and pre-complex formation occurred at high efficiency [47]. According to Figure 5A,B, as the pH values increased, the ionization rate of the MAGA monomer also increased, hence the monomer–analyte interaction increased, which caused an increase in the obtained QCM signals. In particular, the increasing electrostatic interaction between the ionized monomer and the polar groups of the target molecule also improved the sensor sensitivity. As pH values increased from 6.0 to higher pH values, the monomer–analyte interaction decreased because the target molecule became the ionized form and the sensitivity of the QCM sensor decreased. Thus, since the highest QCM signal was obtained at pH 6.0, the subsequent analytical applications were carried out at pH 6.0.

### 3.5. Sensitivity of the MIP/EM/S-g-C_3_N_4_/QCM Sensor

**Figure 6:**
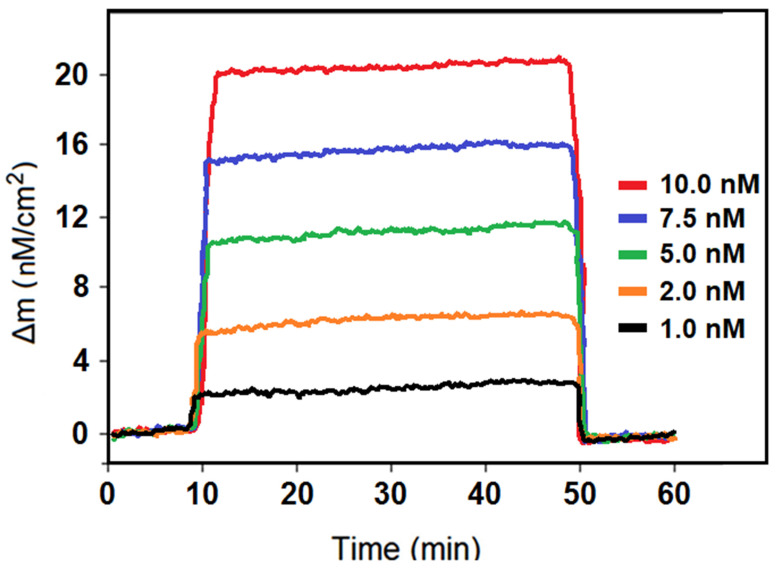
DIM concentration effect on MIP/EM/S-g-C_3_N_4_/QCM in the presence of pH 6.0 PBS (from 1.0 to 10.0 nM DIM).

QCM is a sensor system based on the piezoelectric effect. The production of electrical energy in response to the applied mechanical energy to a material or mechanical energy in response to the applied electrical energy to the material is called the piezoelectric effect. In the QCM technique, where the mass sensitivity depends on the frequency of the crystal and the frequency depends on the thickness of the crystal, the significant analytical applications can be performed depending on the relationship between mass and frequency [48]. In this study, the mass–frequency changes in parallel with the phase density between the MIP/EM/S-g-C_3_N_4_/QCM sensor surface and DIM concentration were recorded instantly and QCM sensorgrams were obtained (Figure 6). The calibration equation of y (Δm) = 1.9842x (C_DIM_, nM) + 0.2608 was obtained using the obtained mass change values against standard DIM solutions including various concentrations. The values of LOQ and LOD were 1.0 × 10^−9^ M and 3.3 × 10^−10^ M, respectively (See Supplementary Materials for the equations). First of all, the molecularly imprinted QCM sensor based on erbium molybdate decorated on sulfur-doped graphitic carbon nitride was successfully developed in the present study and the pesticide analyses have been performed with high sensitivity in comparison with the other methods (Table 1). In addition, it is possible to say that an environmentally friendly sensor design has been achieved by ensuring the production of nanomaterials with high purity under hydrothermal conditions providing minimal waste generation during sensor production. Especially, the production of highly crystallizable nanomaterials has been successfully achieved with the help of this hydrothermal synthesis technique. However, the synthesis of the EM/S-g-C_3_N_4_ nanocomposite at high temperatures and under time-consuming procedures was seen as the only limiting factor. In recent years, both environmental and animal contamination resulting from pesticide exposure has negatively affected healthy life. Thanks to this developed QCM sensor, the analysis of other pesticides such as DIM will be instantly performed with high sensitivity, and early diagnosis of serious diseases resulting from pesticide exposure will be possible. In the literature, it is seen that chromatographic-based analytical methods have been developed for the analysis of the organophosphorus pesticide DIM [49]. However, these techniques are expensive, require expertise, and consume large amounts of chemical agents. Thanks to the developed QCM sensor in this study, such negativities do not occur and an environmentally friendly analysis is performed. As a result, a QCM sensor with instantaneous response time based on the EM/S-g-C_3_N_4_ nanocomposite and a molecularly imprinting polymer with high selectivity, repeatability, and sensitivity has been presented to the world.

### 3.6. Recovery

Recovery experiments were carried out to show that the developed QCM-based sensor could detect DIM in apple juice samples with high accuracy and selectivity. For this purpose, firstly, after the apple juice sample was divided into four equal parts, increasing concentrations of standard DIM solutions were added to all except for the first part. Then, these four parts were diluted to equal volume with PBS (0.1 M, pH 6.0). After DIM analysis of each sample was detected with the developed QCM sensor, the recovery values were calculated. Values close to 100% showed that the designed QCM sensor in this study successfully performed DIM analysis with high selectivity through molecular imprinting technology (Table S1). According to the DIM acceptable value of 8.7 × 10^−8^ M, since the amount of DIM (6.4 × 10^−10^ M) in real apple juice samples was lower than the accepted value, we can say that the used apple juice samples in the study can be consumed safely.

### 3.7. Selectivity, Stability, Reproducibility, and Repeatability of MIP/EM/S-g-C_3_N_4_/QCM

**Figure 7:**
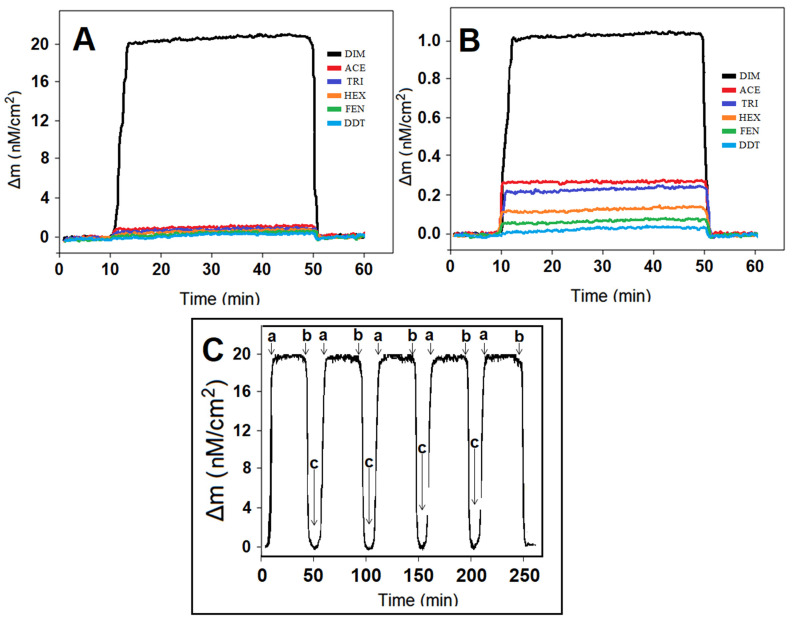
(**A**) QCM sensorgrams of (**A**) MIP/EM/S-g-C_3_N_4_/QCM, (**B**) NIP/EM/S-g-C_3_N_4_/QCM in the presence of 10.0 nM DIM, 1000.0 nM ACE, 1000.0 nM TRI, 1000.0 nM HEX, 1000.0 nM FEN, and 1000.0 nM DDT. (**C**) Repeatability of the MIP/EM/S-g-C_3_N_4_/QCM chip: (a) adsorption; (b) desorption; (c) regeneration.

Molecular imprinting technology provides high selectivity in sensor applications, especially when analyzing real samples. Selectivity tests were carried out to prove the high selectivity of the prepared MIP-based QCM chip in the current study in the presence of other pesticides that would affect the DIM analysis in apple juice samples. To demonstrate the imprinting selectivity of the sensor prepared for this purpose, both MIP-based and NIP-based QCM chips were prepared. The resulting QCM sensorgrams are given in Figure 7A,B. According to the selectivity coefficient (k) and relative selectivity coefficient (k′) values, it appeared that the MIP QCM chip performed DIM analysis with approximately 40 times more selectivity than ACE, approximately 50 times more selectivity than TRI, approximately 67 times more selectivity than HEX, approximately 100 times more selectivity than FEN, and approximately 200 times more selectivity than DDT (Table S2). These results prove that analyses with high selectivity can be performed successfully in apple juice samples.

Stability studies of the developed sensor were carried out by taking continuous QCM sensorgrams of the prepared QCM chip in the presence of 10.0 nM DIM at certain intervals for 6 weeks. The observed QCM signal Δm (nM cm^−2^) at the end of the first week was approximately 97.92% of the observed QCM signal at the end of the sixth week, indicating that the prepared QCM chip had a high stability.

For the reproducibility test of MIP/EM/S-g-C_3_N_4_/QCM, ten different QCM chips were prepared in harmony with Section 2.3 and Section 2.4. The QCM chip signals using ten different QCM chips were calculated in the presence of 10.0 nM DIM and the relative standard deviation (RSD) was 0.27%, suggesting a high reproducibility.

Finally, the repeatability test was performed by completing the five consecutive times “adsorption–desorption–regeneration” cycles in the presence of 10.0 nM DIM. According to Figure 7C, as a result of each cycle, the average RSD value of the obtained QCM signals was 0.81%, indicating a high repeatability.

## 4. Conclusions

In this study, we developed a new molecularly imprinted QCM sensor based on erbium molybdate incorporating sulfur-doped graphitic carbon nitride for rapid dimethoate residue detection. The characterization studies of the synthesized nanocomposite under hydrothermal conditions were successfully carried out using spectroscopic, microscopic, and electrochemical techniques. The QCM sensor exhibited high sensitivity, reproducibility, repeatability, and selectivity, and a new methodology for organophosphorus pesticide residue determination was developed. The linear relationships of the developed QCM sensor showed that this sensor could be utilized for the quantitative detection of dimethoate in the range of 1.0–10.0 nM. Furthermore, this developed QCM sensor did not need expensive instruments. In addition, one of the most serious health risks globally in recent years is the difficult supply of clean and safe foods, especially the intensive use of foods containing pesticides which causes serious health problems such as metabolic diseases. Thus, we can say that the consumption of healthy foods will be possible, especially with the use of the highly selective, sensitive, and easily applicable sensor in this study. Finally, the developed QCM sensor can be utilized in the analysis of various real samples such as vegetables, fruits, and environmental samples.

## Figures and Tables

**Table 1:** Comparison of the prepared MIP sensor with the other novel analytical methods.

Material	Linear Range (M)	LOD (M)	References
Fe-N/C single-atom nanozymes	1.0 × 10^−9^–1.0 × 10^−7^	4.2 × 10^−10^	[50]
Colorimetric biosensor	0.0–1.0 × 10^−5^	1.0 × 10^−6^	[51]
Ag, CuO and Ag-Cu nanoparticles	3.0 × 10^−6^–2.0 × 10^−5^	3.0 × 10^−6^	[52]
Citrate-stabilized AuNPs	1.0 × 10^−9^–4.0 × 10^−8^	2.0 × 10^−8^	[53]
p-sulphonato-calix [4] resorcinarene functionalized silver nanoprobe	1.0 × 10^−7^–1.0 × 10^−6^	8.0 × 10^−8^	[54]
Luminescence turn-on	0.0–1.0 × 10^−4^	1.9 × 10^−5^	[55]
CdSe Quantum Dots	4.5 × 10^−7^–8.0 × 10^−5^	1.3 × 10^−5^	[56]
MIP/EM/S-g-C_3_N_4_/QCM	1.0 × 10^−9^–1.0 × 10^−8^	3.3 × 10^−10^	This study

## Data Availability

The original contributions presented in the study are included in the article and Supplementary Materials, further inquiries can be directed to the corresponding author.

## Supplementary Materials

The following supporting information can be downloaded at: https://www.mdpi.com/article/10.3390/foods13050810/s1, Figure S1: TEM image (A) of the EM/S-g-C_3_N_4_ nanocomposite and HRTEM image (B) of the EM/S-g-C_3_N_4_ nanocomposite; Figure S2: EDX spectrum of the EM/S-g-C_3_N_4_ nanocomposite; Figure S3: (A) CV curves and (B) EIS responses at (a) bare GCE, (b) EM/GCE, (c) S-g-C_3_N_4_/GCE, and (d) EM/S-g-C_3_N_4_/GCE (Redox probe: 1.0 mM [Fe(CN)_6_]^3−/4−^ containing 0.1 M KCl, potential scan rate: 50 mV/s); Figure S4: AFM images of (A) the bare QCM chip, (B) the molecularly imprinted film on EM/S-g-C_3_N_4_/QCM, and (C) FTIR spectrum of molecularly imprinted film on EM/S-g-C_3_N_4_/QCM; Table S1: Recovery results of DIM (*n* = 6); Table S2: k and k′ values of DIM-imprinted QCM chips (MIP/EM/S-g-C_3_N_4_/QCM and NIP/EM/S-g-C_3_N_4_/QCM) (*n* = 6).
