# Supplementary material for: A Novel Molecularly Imprinted Quartz Crystal Microbalance Sensor Based on Erbium Molybdate Incorporating Sulfur-Doped Graphitic Carbon Nitride for Dimethoate Determination in Apple Juice Samples
Source: Foods. 2024 Mar 6;13(5):810. doi: 10.3390/foods13050810 (PMC10930758; doi:10.3390/foods13050810)
Supplement: Supplementary file 1 [file foods-13-00810-s001.zip › foods-2882984-supplementary.pdf]

Supplementary Data

For

A novel molecularly imprinted quartz crystal microbalance sensor based on erbium molybdate incorporating sulphur-doped graphitic carbon nitride for dimethoate determination in apple juice samples

Neslihan Özdemir[1], Betül Karslıoğlu[2], Bahar Bankoğlu Yola[3], Necip Atar[4], Mehmet Lütfi Yola[5*]

[1]*Department of Machinery and Metal Technologies, Merzifon Vocational School, Amasya University, Amasya, 05300, Turkey; neslihan.ozdemir@amasya.edu.tr*
[2]*Department of Gastronomy and Culinary Arts, Faculty of Tourism, Hasan Kalyoncu University, Gaziantep, 27000, Turkey; betul.kokay@hku.edu.tr*
[3]*Department of Engineering Basic Sciences, Faculty of Engineering and Natural Sciences, Gaziantep Islam Science and Technology University, Gaziantep, 27000, Turkey; bahar.bankogluyola@gibtu.edu.tr*
[4]*Department of Chemical Engineering, Faculty of Engineering, Pamukkale University, Denizli, 20160, Turkey; natar@pau.edu.tr*
[5]*Department of Nutrition and Dietetics, Faculty of Health Sciences, Hasan Kalyoncu University, Gaziantep, 27000, Turkey*
**Correspondence: mlutfi.yola@hku.edu.tr; Tel.: +90-3422118080; Fax: +90-3422118081*

Instrumentation

Some analytical devices for the morphological analyzes such as ZEISS EVO 50 SEM (Tokyo, Japan) for field emission scanning electron microscopy (FESEM), JEOL 2100 TEM/HRTEM instrument for transmission electron microscopy (TEM), Rikagu Miniflex, x-ray diffractometer (Tokyo, Japan) for x-ray diffraction analysis (XRD), Bruker-Tensor 27 FTIR spectrometer (Tokyo, Japan) for fourier-transform infrared spectroscopy (FTIR), PHI 5000 Versa Probe type x-ray photoelectron spectrometer (Japan/USA) for x-ray photoelectron spectroscopy (XPS), AFM Park NX10 (Japan) for atomic force microscopy (AFM), Thermo Fisher Scientific UV-Vis/Vis instrumentation for UV-Vis spectra, GAMRY Reference 600 workstation for electrochemical impedance spectroscopy (EIS), and cyclic voltammetry (CV) were applied in present study.

Sensitivity

$$LOQ = 10.0\ S / m$$

$$LOD = 3.3\ S / m$$

S: Standard deviation of the intercept and m*:* Slope of the regression line

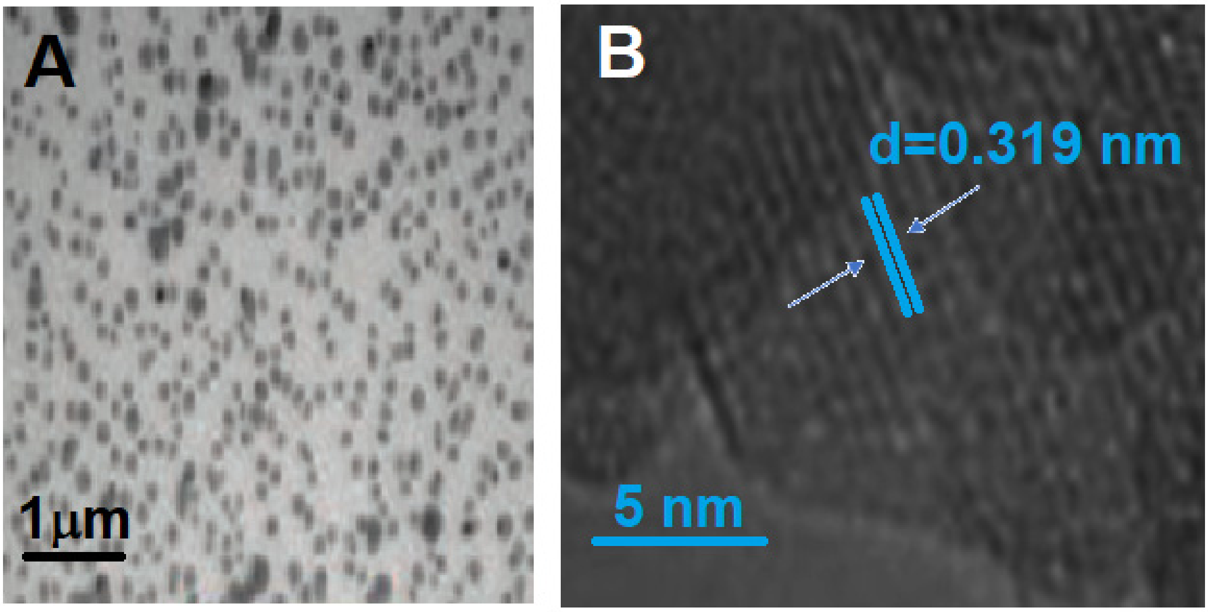

Figure S1. TEM image (A) of EM/S-g-C_3N_4 nanocomposite and HRTEM image (B) of EM/S-g-C_3N_4 nanocomposite

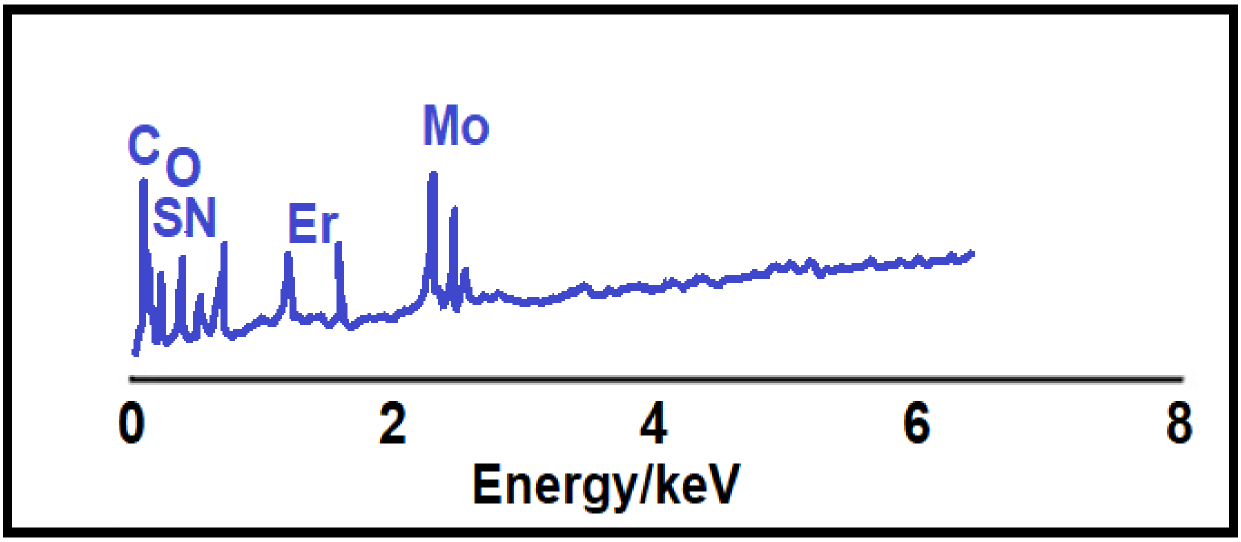

Figure S2. EDX spectrum of of EM/S-g-C_3N_4 nanocomposite

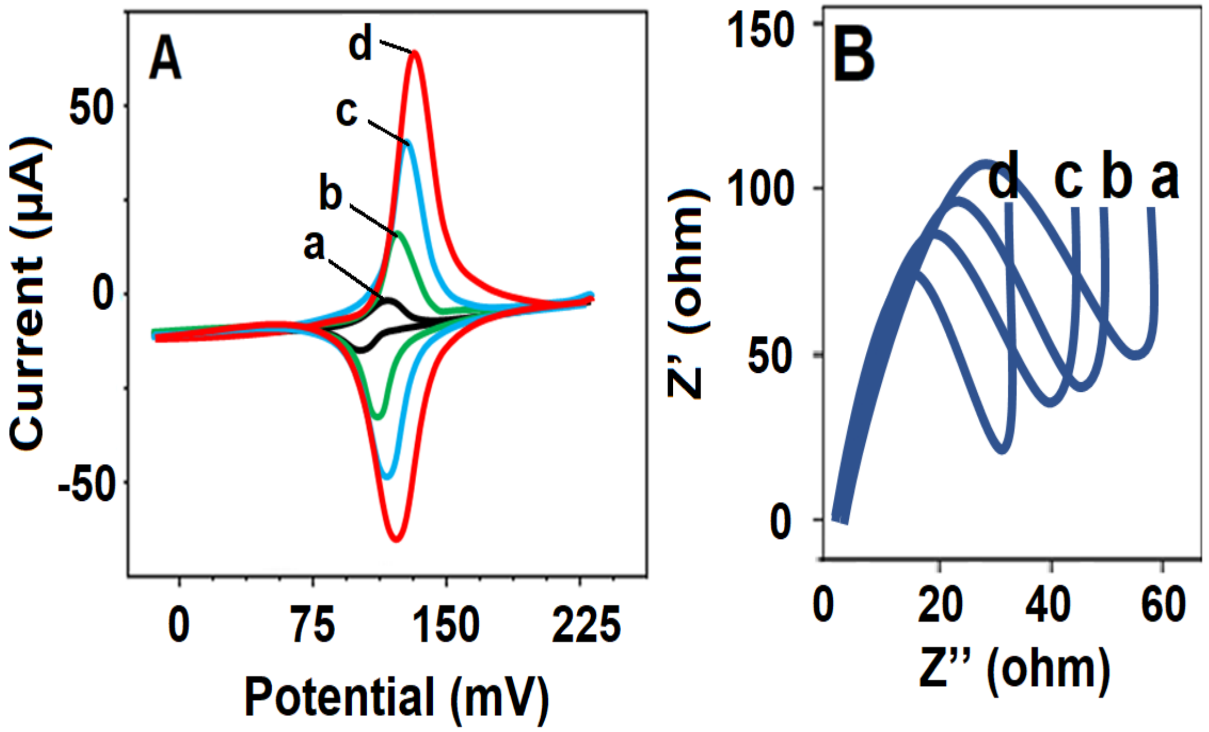

Figure S3. (A) CV curves and (B) EIS responses at (a) bare GCE, (b) EM/GCE, (c) S-g-C_3N_4/GCE, (d) EM/S-g-C_3N_4/GCE (Redox probe: 1.0 mM $[Fe(CN)_6]^{3-/4-}$ containing 0.1 M KCl, potential scan rate: 50 mV/s)

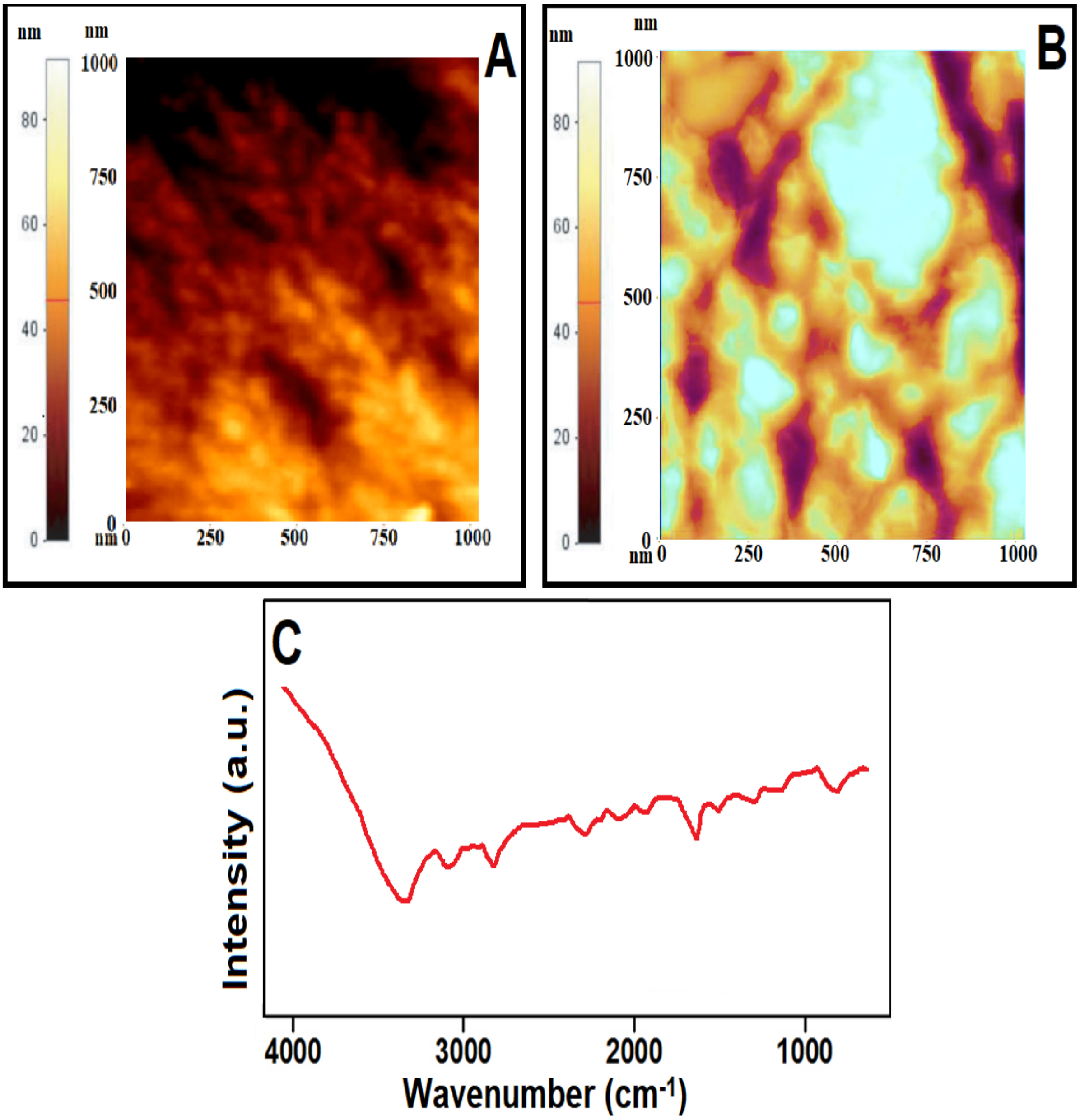

Figure S4. AFM images of (A) bare QCM chip, (B) the molecularly imprinted film on EM/S-g-C_3N_4/QCM and (C) FTIR spectrum of molecularly imprinted film on EM/S-g-C_3N_4/QCM

Table S1. Recovery results of DIM (n=6)

Sample	Added DIM (nM)	Found DIM (nM)	Recovery (%)
Apple Juice	-	0.64 ± 0.01	-
	2.00	2.63 ± 0.03	99.62 ± 0.08
	4.00	4.65 ± 0.09	100.22 ± 0.03
	6.00	6.65 ± 0.07	100.15 ± 0.05

Recovery = Found DIM, nM / Real DIM, nM

Table S2. k and k′ values of DIM imprinted QCM chips (MIP/EM/S-g-C_3N_4/QCM and NIP/EM/S-g-C_3N_4/QCM) (n=6)

	MIP		NIP		
	**Δm (nM cm^{2-})**	**k**	**Δm (nM cm^{2-})**	**k**	**k′**
DIM	20.15 ± 0.01	-	1.05 ± 0.03	-	-
ACE	0.50 ± 0.03	40.30	0.25 ± 0.02	4.20	9.60
TRI	0.40 ± 0.07	50.38	0.20 ± 0.04	5.25	9.60
HEX	0.30 ± 0.08	67.17	0.10 ± 0.07	10.50	6.40
FEN	0.20 ± 0.09	100.75	0.05 ± 0.06	21.00	4.80
DDT	0.10 ± 0.05	201.50	0.01 ± 0.06	105.00	1.92

Analyte concentrations: 10.0 nM DIM, 1000.0 nM ACE, 1000.0 nM TRI, 1000.0 nM HEX, 1000.0 nM FEN, 1000.0 nM DDT

k = $\Delta m_{DIM}/\Delta m_{\text{interfering chemical}}$ and k′ = k_{MIP}/k_{NIP}
